# Simple Blood Test Indicating Sepsis Relapse: A Case Report

**DOI:** 10.7759/cureus.47199

**Published:** 2023-10-17

**Authors:** Barbara Wójcik, Jerzy Superata, Zbigniew Szyguła

**Affiliations:** 1 Department of Clinical Rehabilitation, University of Physical Education in Krakow, Krakow, POL; 2 Department of Sports Medicine and Human Nutrition, University of Physical Education in Krakow, Krakow, POL

**Keywords:** leukocyte, platelets, blood smear, sepsis survivor, sepsis, case report

## Abstract

Delays in the diagnosis and management of sepsis are associated with higher mortality. Moreover, routine blood tests performed just before hospital discharge are still insufficient for sepsis survivors. In this report, for the first time, dramatic hematological changes found in the blood of a sepsis survivor are described. The pictorial information from microscope images associated with an appropriate set of multiparameter laboratory test results enabled for prediction of sepsis relapse four days before its clinical recognition. Thus, the role of this case report is to encourage medical practitioners to introduce (or re-introduce) blood smears as the helpful adjunctive extension of routine blood testing, especially when sepsis is suspected.

## Introduction

Developing new methods to diagnose and monitor sepsis, one of the most elusive and lethal diseases in the world, is a research priority [1,2]. Every year, sepsis affects between 47 and 50 million people, and it is the cause of at least 11 million deaths. Sepsis frequently manifests itself as the clinical worsening of ordinary and avoidable infections, that is, those affecting the respiratory, gastrointestinal as well as urinary tracts, or of wounds and the skin. However, timely and accurate detection still remains a challenge. Sepsis is often not adequately diagnosed at the early stage when it could still be potentially avoidable [2]. In this report, we describe the dramatic hematological changes found in the blood of a sepsis survivor just after discharge from hospital indicating sepsis relapse, yet without symptoms essential for diagnosis according to Sepsis-3 guidelines.

## Case presentation

A 71-year-old man with a history of hypertension and type 2 diabetes, without smoking or drinking habits, was admitted to Ludwik Rydygier Memorial Specialist Hospital in Kraków on July 11, 2018, due to peritonitis. On the same day, laparotomy was performed; purulent content was sucked out of the peritoneal cavity, numerous adhesions were released, two liver abscesses were evacuated, and a ruptured cyst within the head and body of the pancreas was found. During the procedure, the patient demonstrated cardiopulmonary instability and required an infusion of norepinephrine and a transfusion of blood products (two units of packed red blood cells and two units of fresh frozen plasma). At the Intensive Care Unit, post-operative septic shock was diagnosed; therefore, fluid resuscitation and broad-spectrum antibiotics (Tienam - Imipenem/Cilastatin, Vancomycin) were applied. Due to persistent anuria and severe circulatory and respiratory instability, it was decided to start ventilation and continuous dialysis procedures; total parenteral nutrition was also introduced. Thanks to the applied treatment, the patient's condition was stabilized, the infusion of pressor amines was stopped, antibiotic therapy was completed (no positive cultures were found), dialysis was stopped, ventilation settings were reduced, and then the patient was extubated (July 19, 2018). On the next day, feeding via a gastric tube was introduced. On July 21, the patient was transferred to the Department of Surgery, where after healing the wound and removing the drains and the probe (tolerating an oral diet), he was discharged from the hospital in good general condition. The package of blood tests performed at hospital discharge, compared to the results at admission, is presented in Table 1.

**Table 1:** Blood test results performed at hospital discharge, compared to the results at admission.

Test	Reference range	Hospital admission	Hospital discharge
White blood cells	4-10 x 10^3^/μL	50.9	6.48
Erythrocytes	3.9-5.7 x 10^6^/μL	3.21	3.7
Haemoglobin	11-15 g/dL	8.7	10.1
Haematocrit	34-45%	26.5	31.4
Platelets	150-400 x 10^3^/μL	113	317
Sodium	136-146 mmol/L	136	136
Potassium	3.50-5.10 mmol/L	5.48	4.7
Bilirubin	<17 μmol/L	15.8	10.2
Creatinine	72-127 μmol/L	129	53
C-reactive protein	<0.5 mg/L	199	113

Just after discharge (August 3, 2018), the patient was frail but reported feeling good, and he was qualified to participate in the RehaSep Project dedicated for sepsis survivors [3]. Thus, according to the study protocol, extensive laboratory tests were ordered at the hospital laboratory (August 6, 2018) and showed leukocytosis (26,950/mm^3^) with 89.2% neutrophils, 7.5% lymphocytes, 2.5% monocytes, increased immature granulocyte percentage count (0.6%), normal platelet count (395,000/mm^3^), decreased hemoglobin (10.1 g/dL) and hematocrit (31.8%) in the automated differential blood count, increased C-reactive protein (189.6 mg/L) and prothrombin time (15.2 sec), partial thromboplastin time (30.2 sec), fibrinogen (3.4 g/L), lactate (3.32 mmol/L), and decreased albumin (19 g/L). A full picture of the results is presented in Table 2.

**Table 2:** Results of extensive blood tests (appropriate set of labs dedicated for sepsis survivors). aPTT, activated partial thromboplastin time; INR, international normalized ratio; ALT, alanine transaminase; AST, aspartate transferase; GGTP, gamma-glutamyl transpeptidase

Test	Reference range	Results
White blood cells	4.0-10 x 10^3^/μL	26.95
Neutrophils	1.6-7.0 x 10^3^/μL	24.04
	40-70%	89.2
Lymphocytes	0.8-4.5 x 10^3^/μL	2.01
	20-45%	7.5
Monocytes	0.20-1.20 x 10^3^/μL	0.68
	5.0-12%	2.5
Eosinophils	0.02-0.60 x 10^3^/μL	0.00
	0.4-6.0%	0.00
Basophils	0.00-0.10 x 10^3^/μL	0.05
	0.1-1.2%	0.2
Immature granulocytes	0.00-0.03 x 10^3^/μL	0.17
	0.0-0.5%	0.6
Erythrocytes	3.9-5.7 x 10^6^/μL	3.71
Hemoglobin	12.0-17.0 x g/dL	10.1
Hematocrit	40.0-51.0%	31.8
Retikulocytes	0.50-2.50%	1.05
Platelets	150-400 x 10^3^/μL	395
aPTT	25.4-36.9 sek	30.2
Fibrinogen	2-4 g/L	3.4
Prothrombin ratio	80-120%	76.5
INR	2-4% (therapeutic range)	1.3
Prothrombin time	9.4-12.5 sek	15.3
Sodium	136-146 mmol/L	135
Potassium	3.50-5.10 mmol/L	3.64
Chloride	98-106 mmol/L	99
Magnesium	0.73-1.06 mmol/L	0.57
Urea	2.5-6.6 mmol/L	6.5
Creatinine	72-127 μmol/L	70
Uric acid	208-428 μmol/L	308
ALT	<40 U/L	13
AST	<42 U/L	22
Bilirubin	<17 μmol/L	8.9
GGTP	7-50 U/L	60
Albumin	35-52 g/L	19
C-reactive protein	<5 mg/L	189.6
Calcium	2.0-2.6 mmol/L	2.03
Lactate	0.5-22 mmol/L	3.32
Vitamin D 25 OH	30.0-80.0 ng/mL	16.8

**Figure 1:**
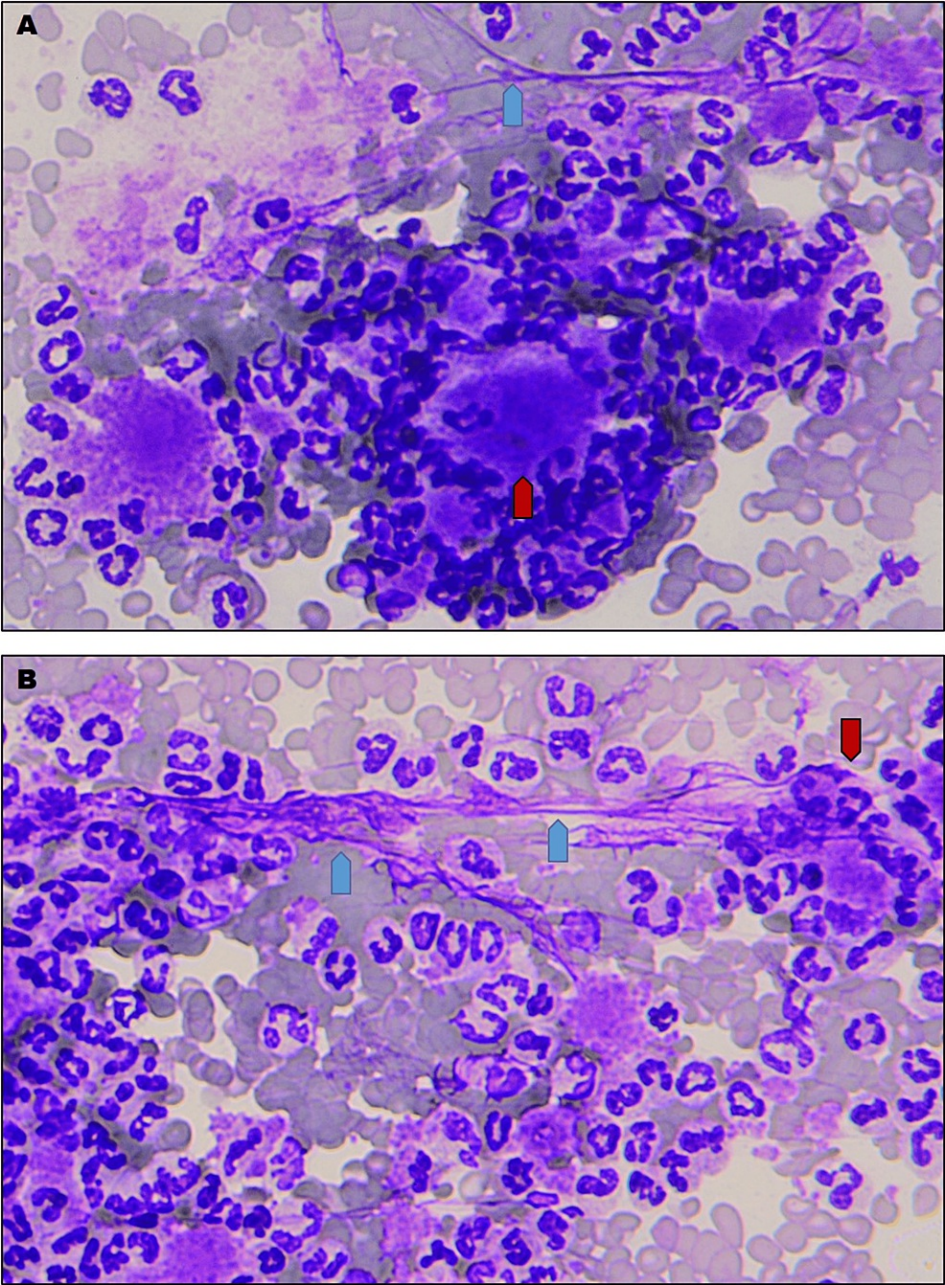
Peripheral (EDTA) blood buffy-coat smear from sepsis survivor just after hospital discharge A-B demonstrating increased platelet activation associated with platelet-leukocyte aggregates (red arrowheads) and fibrous deposits (blue arrowheads), magnification x200

The additional, manually performed peripheral blood buffy-coat smear with the Hemacolor Rapid stain (Merck) showed an image of excessive platelet activation associated with large platelet-leukocyte aggregates and fibrous deposits (Figure 1 a-b). However, only after four days, when the patient’s condition worsened, he was re-admitted to the hospital due to secondary peritonitis and intra-abdominal sepsis, which he survived, but died five months after as a result of cachexia and pneumonia.

## Discussion

It is becoming increasingly apparent that excessive activation of platelets is one of the key events in the development of sepsis. Beyond their important role in hemostasis, platelets are crucial regulators of leukocyte function and, thus, of inflammatory immune responses [4,5]. Platelets, by changing the surface expression of P-selectin and CD40, which are recognized by leukocyte P-selectin glycoprotein ligand-1 and CD154, can directly interact with circulating leukocytes, leading to the formation of platelet-leukocyte aggregates (PLAs). Moreover, increased P-selectin expression by activated platelets enhances the generation of platelet microparticles that are proinflammatory, procoagulant membrane vesicles with a great capacity to develop adhesive interactions with diverse cell types and promote activation of endothelial cells, leukocytes, and other platelets [6].

During the process of immunothrombosis, platelets and immune cells form a physical barrier of confinement to limit pathogens' systemic spread in the bloodstream. However, excessive and dysregulated activation of platelets results in tissue ischemia due to the development of thromboinflammation [7].

Sepsis is a multifaceted process, and even though the symptoms may improve, the downturn can happen very quickly. While protective humoral and cellular immune responses in good health status (homeostasis) have the maximum effectiveness against a pathogen, immune responses in susceptible patients are frequently impaired and loss of balance may persist for a long time [8].

Dysregulated cellular reactions with concomitant uncontrolled activation of the complement and coagulation influence inflammatory tissue damage and the septic process may progress often before characteristic clinical symptoms become clearly visible [9]. However, according to Sepsis-3 guidelines, sepsis, defined as a “life-threatening organ dysfunction caused by a dysregulated host response to infection”, is basically diagnosed by the Sequential Organ Failure Assessment (SOFA) score, often too late because each passing hour increases the risk of death [10].

An increased level of circulating PLAs is observed already at an early phase of sepsis [9,11]. However, in non-survivors and patients developing multiple organ failure, such changes in blood samples are significantly decreased likely due to enhanced peripheral sequestration, which manifests as apparent sepsis-associated thrombocytopenia [9,11]. Therefore, repeated examination of the peripheral blood smear or video microscopic assessment of microcirculation is recommended during the course of a sepsis episode [12,13].

For the first time, we report that significant hematological changes can be seen well before the recognizable sepsis symptoms appear. This evidence would imply that peripheral blood buffy-coat smear examination may become an additional simple and useful tool for improving the diagnosis of sepsis and/or warning of relapse.

Our findings reported here raise a number of questions that could be addressed in future studies of similar cases. How characteristic are the observed images for the developing sepsis process? Are they reproducible/comparable among patients? What is the time course of blood image changes? Could blood cell image changes always precede clinical symptoms? More cases should definitely be investigated as we have not yet observed such dramatic and characteristic changes in hematological images in other sepsis survivor patients examined in the same way, despite evidently increased inflammatory parameters of some of them. However, none of them was affected by sepsis relapse.

## Conclusions

Our research results suggest that the routine blood tests performed just before hospital discharge are still insufficient for sepsis survivors. They require extended, multiparameter monitoring including, in particular, indices related to the immune system status as well as imaging data. Therefore, intensive efforts should be made to further develop cell analysis methods better applicable to medical diagnostics.

The teaching role of this case report is to encourage medical practitioners to introduce (or re-introduce) blood smears as the helpful adjunctive extension of routine blood testing, especially when sepsis is suspected.
